# Therapeutic effects of maximal strength training on walking efficiency in patients with schizophrenia – a pilot study

**DOI:** 10.1186/1756-0500-5-344

**Published:** 2012-07-03

**Authors:** Jørn Heggelund, Gunnar Morken, Jan Helgerud, Geir E Nilsberg, Jan Hoff

**Affiliations:** 1 Faculty of Medicine, Department of Neuroscience, Norwegian University of Science and Technology, Trondheim, Norway; 2 Division of Psychiatry, Department of Research and Development (AFFU), St. Olavs University Hospital, Trondheim, Norway; 3 Division of Psychiatry, Department of Østmarka, St. Olavs University Hospital, Trondheim, Norway; 4 Faculty of Medicine, Department of Circulation and Medical Imaging, Norwegian University of Science and Technology, Trondheim, Norway; 5 Hokksund Medical Rehabilitation Centre, Hokksund, Norway; 6 Department of Sports and Outdoor Life Studies, Telemark University College, Bø, Norway; 7 Department of Physical Medicine and Rehabilitation, St.Olavs University Hospital, Trondheim, Norway

**Keywords:** Exercise, Gait disturbances, Walking efficiency, Schizophrenia

## Abstract

**Background:**

Patients with schizophrenia frequently have disabling gait deficits. The net mechanical efficiency of walking (*ϵ*_*net*_) is an accurate measure often used to evaluate walking performance. Patients with gait deficits have a reduced *ϵ*_*net*_ with excessive energy expenditure during sub-maximal walking. Maximal strength training (MST) improves *ϵ*_*net*_ in healthy individuals and is associated with reduced risk of mortality. The aim of this study was to investigate whether MST improves *ϵ*_*net*_ in patients with schizophrenia.

**Methods:**

Patients (ICD-10 schizophrenia, schizotypal or delusional disorders (F20-F29)) were included in a non-randomized trial. Patients were assigned to one of two groups: 1) MST consisting of 4x4 repetitions at 85-90% one repetition maximum (1RM) performed in a leg press apparatus or 2) playing computer games (CG). Both groups carried out their activity three days per week for eight weeks. 1RM, *ϵ*_*net*_ at 60 watt walking, peak oxygen uptake (VO_2peak_), the Positive and Negative Syndrome Scale (PANSS) and the 36-items short form (SF-36) were measured pre and post intervention.

**Results:**

The baseline *ϵ*_*net*_ was 17.3 ± 1.2% and 19.4 ± 3.0% in the MST (n = 6) and CG groups (n = 7), respectively, which is categorized as mechanical inefficiency. The MST group improved 1RM by 79 kg (*p* = 0.006) and *ϵ*_*net*_ by 3.4% (*p* = 0.046) more than the CG group. The MST group improved 1RM and *ϵ*_*net*_, by a mean of 83 kg (*p* = 0.028) and 3.4% (*p* = 0.028), respectively. VO_2peak_ at baseline was 34.2 ± 10.2 and 38.3 ± 9.8 ml·kg^-1^·min^-1^ in the MST and CG groups, respectively, and did not change (*p* > 0.05). No change was observed in PANSS or SF-36 (*p* > 0.05).

**Conclusions:**

MST improves 1RM and *ϵ*_*net*_ in patients with schizophrenia. MST could be used as a therapeutic intervention for patients with schizophrenia to normalize their reduced *ϵ*_*net*_.

## Background

Patients with schizophrenia frequently have disabling gait deficits. Often the patients have decreased walking speed, reduced stride length and balance deficits [1,2]. Gait deficits occur independently of the side effects of antipsychotic medication, but conventional antipsychotic treatment seems to worsen it [1]. Gait deficits influence the patients’ well-being, quality of life and compliance to antipsychotic treatment [3,4].

Net mechanical efficiency of walking (*ϵ*_*net*_) is a common physiological method used to evaluate walking and the metabolic consequences of gait deficits. This measure determines the magnitude of metabolic energy expenditure converted into mechanical work during walking. Gait deficits such as those in Parkinson’s disease increase the metabolic cost of walking and reduce *ϵ*_*net*_[5]*.* Consequently, patients with poor *ϵ*_*net*_ use excessive amounts of energy to sustain a given sub-maximal workload, which contributes to an elevated level of physical fatigue. Findings from our lab indicate that *ϵ*_*net*_ is reduced in patients with schizophrenia before entering an endurance training program [6].

Patients with poor *ϵ*_*net*_, such as those with coronary artery disease and peripheral arterial disease, have derived benefit from lower limb maximal strength training (MST) [7-9]. MST aims to increase the ability to lift the highest possible weight in one repetition (1RM) and the ability to produce high forces at great speed. These adaptations have shown significant impacts in rehabilitation programs owing to their carry-over effects on walking performance measured as *ϵ*_*net *_[8,10,11]. MST also has a number of beneficial effects, such as increased basal metabolic rate, improved glucose metabolism and improved cholesterol levels, that could reduce the high risk of cardiovascular disease and metabolic syndrome in patients with schizophrenia [12,13]. Strength training is scarcely investigated in patients with schizophrenia and no studies have investigated the effects of strength training on *ϵ*_*net*_ in patients with schizophrenia.

The objective in this pilot-study was to evaluate effects of 8 weeks MST on 1RM, *ϵ*_*net*_, symptoms of schizophrenia and quality of life. We chose to compare the MST with inactivity in the form of playing a computer game (CG) over the same number of sessions. We hypothesized that MST would improve 1RM and *ϵ*_*net*_ more than inactivity (CG).

## Methods

### Study design

A controlled pilot study with pre-post intervention design was carried out. One group performed MST and one group spent the same amount of time playing computer games (CG). A non-randomized trial design was incorporated in this pilot study in order to prevent distrust of patients due to randomization and to avoid excluding the most severely ill patients [14]. The sample size estimate was based on the results of mechanical efficiency from a similar study showing a 3.4% mean difference in change between groups with a 95% CI (−0.01 to 6.8). The change was significant (*p* < 0.05) using a Mann–Whitney U test and 6 patients in each group [10]. The CG group also serves as a control group in another study [6]. All included patients were considered suitable for participation in both interventions. We first included patients to the CG group and then to the MST group. All patients performed pre and post tests and a supervised intervention three times per week in 8 weeks. The outcome assessment was not blinded. To avoid experimenter bias the planned test protocol was exactly adhered to in both groups.

### Patients

Patients were in- and outpatients at the University Hospital psychiatric department. All patients lived under supervised conditions. To be included, patients had to have ICD-10 schizophrenia, schizotypal or delusional disorders (F20-F29). Patients were excluded if they had coronary heart disease, chronic obstructive pulmonary disease, any major changes in pharmacological treatment during the intervention period, were unable to perform testing, or adhered to less than 19 training sessions (80%). Included patients did not perform strength training or play computer games outside of the study. The patients’ traditional therapy was unchanged during the intervention period. All patients took neuroleptics (Table 1). To clarify inclusion/exclusion criteria and somatic health a medical doctor performed a health examination at the beginning of the study. Diagnoses were confirmed by medical records. Sixteen patients were included, the first nine were assigned to CG and the next seven patients were assigned to MST. There were 5 in-patients included in the study, all of them in the CG group. Three patients did not complete the training period: in the CG group one patient was discharged from the hospital before completion and one patient disappeared; in the MST group, one patient was excluded because the patient completed less than 80% of the training sessions. No acute psychotic episodes were registered during the intervention. The study was approved by the National Committee for Medical and Health Research Ethics (REK Midt) and was conducted according to the Helsinki declaration. Written informed consent was obtained from all the included patients after the procedures were fully explained. Characteristics of the patients are presented in Table 1.

**Table 1:** **Characteristics of the included patients**^**a**^

	**Maximal strength training (N = 6)**	**Computer game training (N = 7)**	**Between groups**^**b**^***p***
Male/Female, N	1/5	4/3	0.266^c^
Age, year	37.5 ± 9.6	38.9 ± 11.4	1.000
Age at first contact with psychiatric services, year	23.5 ± 7.7	25.2 ± 6.3	0.836
Months of hospitalization	107.5 ± 58.6	70.0 ± 62.9	0.366
Height, cm	162 ± 10	175 ± 10	0.051
Body weight pre-intervention, kg	81.2 ± 32.2	85.3 ± 29.7	1.000
Body weight post-intervention, kg	82.5 ± 30.8	87.1 ± 29.9	0.945
Body mass index pre-intervention, kg·m^-2^	30.7 ± 11.2	27.6 ± 8.5	0.628
ICD-10 diagnosis, N			
Paranoid schizophrenia	2	4	0.592^c^
Disorganized schizophrenia	1		
Undifferentiated schizophrenia	2	2	
Unspecified schizophrenia	1		
Schizoaffective disorder		1	
Use of neuroleptics, N			
Clozapine	4	6	0.559^c^
Clozapine combined with neuroleptics	3	4	
Clozapine combined with risperidone	1		
Risperidone		1	
Zuclopenthixol	1		
Haloperidol	1		

^a^Data is presented as mean ± standard deviation.

^b^Man-Whitney U Test.

^c^Fisher’s exact test.

### Interventions

All MST sessions started with a five minute warm-up on the treadmill at a workload corresponding to 70% of peak heart rate (HR_peak_; the highest heart rate measured during the last minute of the peak oxygen uptake (VO_2peak_) test). MST was then performed at a 54˚ incline leg press machine (Technogym, Italy). The weight was lowered in a controlled manner in the eccentric phase until the patient reached 90 degrees in the knee joint. Then the patients had a short stop (~0.5 second) before the weight was moved as rapidly as possible to complete extension. The training volume was four sets of four repetitions with a load corresponding to 85-90% 1RM. An exercise physiologist supervised the training sessions and ensured that the training load was increased with 2.5-5 kg each time patients managed to perform four sets with the determined load. Rest periods were three minutes between each set.

The CG group spent 36 minutes three times per week training to improve their ability in the computer game Tetris (THQ Inc. Calabasas Hills, CA), using an Xbox Video Game Systems (Microsoft Corporation, Redmond, USA). All sessions were monitored and patients were encouraged to improve performance.

Interventions were performed in the hospital’s physical training facilities, with easy access for patients and staff. MST and CG was performed in separate rooms. Members of the staff were dedicated to implement the interventions as an integrated part of the treatment program.

### Outcome measurements

Physical testing started with a ten minute warm-up at the treadmill at approximately 50-60% of VO_2peak_. Patients then performed a six minute sub maximal walk on the DKCity treadmill (Tung Keng Enterprise CO., LTD. Taiwan) at a load corresponding to 60 watt (60 Newton meters per second, 60 N · m · sec^-1^) and without holding onto the handrails. The speed and inclination at 60 watt was calculated from the bodymass (m_b_) in kilo and elevation on the treadmill using Equation (1). The speed range used in the calculation was three to six kilometers per hour (km·h^-1^) and the elevation was adjusted accordingly. Sin θ is the sinus to the angle of elevation on the treadmill.

(1) km·h−1=60N·m·sec−1mb·9·81N·sinθ°·3·6km·h−1

The measurements of pulmonary gas exchange were obtained between 5 and 5.5 minutes walking, using the Cortex Metamax II portable metabolic test system (Cortex Biophysik GmbH, Leipzig, Germany). Heart rate was assessed concurrently using a Polar S610i heart rate monitor (Polar Electro, Finland). *ϵ*_*net*_ was determined as the percentage of the work input (kilocalories) that is converted into work output, as presented in Equation (2). $\dot{W}$ is the steady power produced during walking, expressed as the Watt production converted into kilocalories per minute (Watt · 0.01433 Kcal·min^-1^). The net metabolic rate (*Ė*_*net*_) is kilocalories per minute estimated from the net oxygen consumption and the caloric value of the corresponding respiratory quotient (RQ). The net oxygen consumption is calculated by deducting the estimated resting oxygen consumption (3.5 mL·kg^-1^·min^-1^) and the horizontal component of walking (0.1 mL·kg^-1^·min^-1^) [15] from the total oxygen consumption measured during walking.

(2) $$\epsilon_{\text{net}}=\frac{\dot{W}}{{\dot{E}}_{\text{net}}}\cdot 100$$

Immediately after testing *ϵ*_*net*_, the patients proceeded with the VO_2peak_ testing protocol. The speed or the inclination was increased every minute until the patient was no longer able to continue, preferentially within 3–6 min. VO_2peak_ was accepted when VO_2_ leveled off despite further increases in speed and when respiratory exchange ratio (RER) was above 1.10 [16]. The highest heart rate recorded during the last minute of the test was determined as HR_peak_.

1RM was measured on the leg press machine using Olympic weights (Eleiko, Sweden). The lift was performed from complete extension to 90 degrees angle in the knee joint and back to complete extension. The load was increased successively until the patient was not able to lift the weight. Test procedures were carefully explained to the patients.

Symptoms of schizophrenia were assessed with the Positive and Negative Syndrome Scale (PANSS) [17]. The 36-item short form (SF-36) self-report instrument was used to assess the physical health and mental health aspect of health related quality of life [18]. SF-36 scales are found to be reliable for patients with schizophrenia [19].

### Statistical methods

Data are expressed as mean and standard deviations (*SD*) unless otherwise noted. A related samples Wilcoxon test was used to determine changes from pre to post intervention. The Independent Sample Mann–Whitney U test was used to evaluate the changes and differences between groups. Categorical variables were tested using Fisher’s exact test. The significance level was set at *p* < 0.05 (2-tailed). Statistical analyses were performed using the software program SPSS, version 17.0 (Statistical Package for Social Science, Chicago, IL).

## Results

Patients in the MST (n = 6) and CG (n = 7) groups performed a mean of 85 ± 9% and 83 ± 6% of the scheduled training sessions, respectively. One patient in the CG group refused to execute maximal strength testing in the leg press machine. Apart from this missing test, the patients performed all of the planned tests.

**Figure 1:**
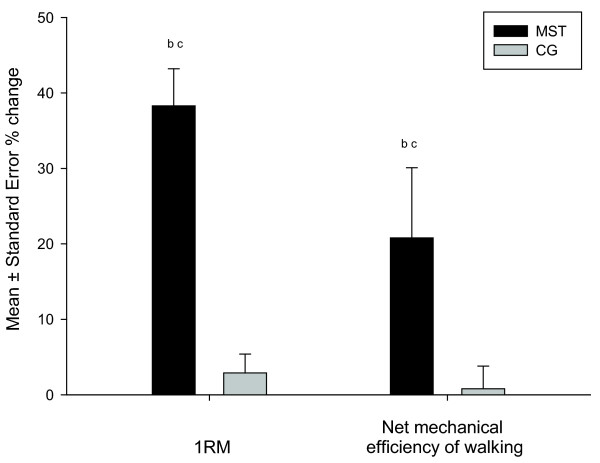
**Percent changes in one repetition maximum (1RM) and net mechanical efficiency of walking.** MST, maximal strength training; CG, playing computer games. ^b^differences from pre to post intervention (*p* < 0.05). ^c^differences in change between groups (*p* < 0.05).

The two groups did not differ pre-intervention with regard to 1RM (*p* = 0.357), *ϵ*_*net*_ (*p* = 0.063), VO_2peak_ (*p* = 0.253), total PANSS (*p* = 0.517) and SF-36 scores (physical health score: *p* = 0.749, mental health score: *p* = 0.200). During eight weeks of training, the MST group improved mean 1RM by 79 kg more than the CG group (*p* = 0.006). The MST group improved 1RM with 83 kg (*p* = 0.028), from mean 218 ± 45 kg to 301 ± 65 kg while the CG group did not change from pre- 214 ± 100 kg to post-intervention 218 ± 97 (*p =* 0.465). *ϵ*_*net*_ improved by 3.4% more in the MST group than in the CG group (*p* = 0.046; Table 2). The improvement in *ϵ*_*net*_ within the MST group was 3.4% (*p* = 0.028; Table 2). No change was observed in the CG group (*p >* 0.999). Changes in 1RM and *ϵ*_*net*_ from pre- to post-intervention are presented as percent changes in Figure 1. The VO_2_ cost of walking at 60 watt was also reduced for the MST group after the intervention (*p* = 0.028), but not for the CG (*p* = 0.0176; Table 2). The VO_2_ reduction in the MST group was not significantly larger than in the CG (*p* = 0.063), but was supported by a grater reduction in pulmonary ventilation (*p* = 0.032) and the respiratory exchange ratio (*p* = 0.031) in the MST group. No changes were observed from pre to post-intervention in VO_2peak_ in either group or between the groups (*p* > 0.05). Pre- and post-intervention VO_2peak_ was 34.2 ± 10.2 and 32.3 ± 8.5 ml·kg^-1^·min^-1^ in the MST and 38.3 ± 9.8 and 37.9 ± 9.9 ml·kg^-1^·min^-1^ in the CG group.

**Table 2:** **Physiological variables measured during 60 Watt constant load sub-maximal treadmill walking**^**a**^

	**Maximal strength training (n = 6)**		**Computer game training (n = 7)**		**Difference pre-post between groups**
	**Pre**	**Post**	**Pre**	**Post**	**Mean (SE)**
Net mechanical efficiency of walking (*ϵ*_*net*_), %	17.3 ± 1.2	20.7 ± 2.9 ^b^	19.4 ± 3.0	19.4 ± 2.5	3.4 (1.6) ^c^
Oxygen uptake					
L·min^-1^	1.71 ± 0.38	1.60 ± 0.37 ^b^	1.77 ± 0.49	1.77 ± 0.45	−0.12 (0.06)
ml·kg^-1^·min^-1^	22.0 ± 3.1	20.0 ± 2.9 ^b^	21.5 ± 3.5	21.1 ± 3.2	−1.5 (0.7)
HR, beats·min^-1^	143 ± 9	137 ± 12	136 ± 17	133 ± 11	−4 (5)
V_E_, L·min^-1^	44.9 ± 12.2	39.9 ± 12.2 ^b^	45.5 ± 15.7	44.8 ± 12.8	−4.4 (1.7) ^c^
RER	0.95 ± 0.06	0.90 ± 0.06 ^b^	0.93 ± 0.04	0.92 ± 0.04	−0.04 (0.01) ^c^

^a^Data is presented as mean ± standard deviation. HR: heart rate, V_E_: total pulmonary ventilation, RER: respiratory exchange ratio, SE: standard error.

^b^differences within groups from pre to post intervention (*p* < 0.05).

^c^differences in change between groups (*p* < 0.05).

No significant changes were observed from pre- to post-intervention in the two groups in total PANSS (MST: *p* = 0.115, CG: *p* = 0.753), SF-36 physical health score (MST: *p* = 0.173, CG: *p* = 0.116) or mental health score (MST: *p* = 0.463, CG: *p* = 0.345). There were no differences in changes between groups (total PANSS: *p* = 0.086, physical health score: *p* = 0.688, mental health score: *p* = 0.262). The pre-intervention mean total PANSS score was 58 ± 12 and 63 ± 20 in the MST and CG groups respectively. The mean post-intervention PANSS scores was 68 ± 8 and 61 ± 19, in MST and CG groups respectively. The SF-36 mean physical health scores at pre- and post- intervention, were 45 ± 10 and 51 ± 11 in the MST group and 46 ± 8 and 48 ± 6 in the CG group, respectively. The mean mental health scores at pre- and post-intervention, were 36 ± 5 and 35 ± 8 in the MST group and 45 ± 11 and 48 ± 2 in the CG group, respectively.

## Discussion

The findings indicate that patients with schizophrenia could safely participate in maximal strength training with a beneficial improvement in maximal strength and walking performance due to improved *ϵ*_*net*_. These improvements occurred without affecting symptoms of schizophrenia.

The MST group improved their 1RM in incline leg press by 38%. This level of improvement is comparable with findings from studies investigating MST in patients with heart disease, chronic obstructive pulmonary disease, healthy subjects and distance runners. Improvements ranging from 27 to 44% have been reported after 8 weeks [8,10,11,20]. In line with the findings of Auchus et al. [21] this study suggests that patients with schizophrenia are able to participate in strength training and improve their 1RM. The improvement in 1RM occurred without changes in bodyweight.

The MST group improved *ϵ*_*net*_ significantly more than the CG group. This is in agreement with the strength training studies that have addressed effects on endurance performance. Although clinically significant, the 20% improvement in *ϵ*_*net*_ is somewhat smaller compared to 32-35% improvement found in other patient groups [8,10]. This might indicate that factors associated with the illness itself or side effects from antipsychotic medication may hamper the transfer effect from improved 1RM to improved *ϵ*_*net*_. As expected, the VO_2peak_ did not change. Maximal strength training mainly results in neural system adaptations with improved maximal strength and ability to develop speed. MST does not result in cardiovascular system changes such as increased peak stroke volume and increased VO_2peak _[7]. However, increased VO_2peak_ from endurance training is often followed by improved *ϵ*_*net *_[6]. In this respect, the improved *ϵ*_*net*_ in this study is considered being a result of the MST intervention, not confounded by increased VO_2peak_. On the contrary, the mean VO_2peak_ in the MST group worsened in absolute terms. The most plausible explanation is that patients could have reduced their endurance physical activities during the intervention. No outside intervention activity control were carried out.

The initial *ϵ*_*net*_ of 17.3% (MST) and 19.4% (CG) can be considered low and within the range of patients that walk with mechanical inefficiency [8,22]. The *ϵ*_*net*_ values in the present study are similar to values found in patients with coronary artery disease (19.2%) and lower than in healthy controls (24.7%) [22]. Gait deficits in schizophrenia have been reported, with the poorest gait patterns found in patients treated with conventional antipsychotics [1]. Patients with schizophrenia choose to walk slower (2.7 to 3.1 km·h^-1^), compared to healthy controls (3.4 km·h^-1^) [1]. From a metabolic point of view, it seems natural that the patients would walk slower if they had lower VO_2peak_ and poorer *ϵ*_*net*_. Walking is usually undertaken at a speed that coincides with the lowest metabolic cost. Poor *ϵ*_*net*_ might be an obstacle in performing daily tasks and so might prevent patients from engaging in activities that require walking. Additionally, poor *ϵ*_*net*_ as well as other gait deficits might have implications for the patients’ well-being [3]. Of interest, the MST improved *ϵ*_*net*_ to a level that is closer to healthy individuals [8,22].

Our study revealed no changes in the total PANSS score or SF-36. In absolute terms, there was an increase of 10 points in the MST group and a decrease of 2 points in the CG group on the total PANSS score. As there are no reasons to anticipate an adverse effect from MST, as would be reflected by an increase in PANSS score, we believe this tendency of a change is due to natural variations in the course of the illness and is probably valid for patients with schizophrenia. There is a small sample size in this study and other mediators or moderators such as depression, anxiety or fatigue due to the intervention were not measured. There are few reports of strength training on symptoms of schizophrenia. A single-group study found that patients reported improvement in a questionnaire that measured perceived psychological well being, but no improvement in the Zung Self Rating Depression Scale (SDS), after weight lifting therapy [21]. A case study described some improvements in Brief Psychiatric Rating Scale (BPRS) and the Nurses’ Observational Scale for In-Patient Evaluation (NOSIE 30). The patient showed beneficial behavior changes in his daily activities. However, there is a need for larger scale and longitudinal intervention studies to determine whether strength training has the ability to decrease symptoms of schizophrenia. Considering the effect of MST on *ϵ*_*net*_ this seems to be sufficient reason to include MST in the treatment of schizophrenia. Furthermore, patients with schizophrenia have a high risk of dying from cardiovascular disease [23]. The 8-weeks training period performed in this study may not be sufficient to affect traditional measures of cardiovascular disease. Further studies should consider a longer period to investigate effects on common blood values. Patients who maintain a certain level of maximal strength through life may have a better chance of sustaining good health. A prospective cohort study of 8762 men aged from 20–80 years, found that maximal strength was independently and inversely associated with all-cause mortality [24]. The association persisted even after adjusting for cardiorespiratory fitness.

There are some limitations in this study. First, the allocation of patients was not randomized. Thus, a selection bias cannot be ruled out. To reduce the selection bias we ensured that all patients were eligible for both interventions. None of the patients were selected because they fitted one of the groups more than the other. Patients were not able to choose a particular group, but their personal preference for the intervention they were asked to participate in could have influenced their decision. A possible disadvantage of the non-randomized design is disclosed in the fact that the MST tend to be hospitalized longer, younger at first contact with psychiatric services and include more women. It could be hypothesized that both severity of illness and gender influenced baseline characteristics such as the SF-36, *ϵ*_*net*_ and VO_2peak_. On the contrary, the effects of the interventions might not be very different between genders. However, the objective of this study was to evaluate the effects of the intervention those might not be different between genders. Although the outcome assessments were not blinded, all tests followed strict procedures and patients seemed to be motivated to perform their best regardless of the group. The effect of the intervention can only be generalized to patients that adhere to MST. The study also had a small sample size. This might be one reason for the tendency of a worsening of PANSS symptoms in the MST group. Furthermore, the 8-week intervention might be too short a period of time for patients suffering from schizophrenia to adapt to the intervention.

## Conclusions

The current study found that the MST improved the patients 1RM to the same level as has been observed in other patient groups and in healthy controls. The MST did also improve the patients’ ability to normalize the net mechanical efficiency of walking. MST is a safe and effective intervention to improve 1RM and net mechanical efficiency of walking in patients with schizophrenia.

## Abbreviations

CG: Computer games; HR_peak_: Peak heart rate; m_b_: Bodymass; MST: Maximal strength training; N · m · sec^-1^: Newton meters per second; PANSS: Positive and Negative Syndrome Scale; RER: Respiratory exchange ratio; SF-36: 36-item short form; Sin θ: Sinus to the angle of elevation on the treadmill; VO_2peak_: Peak oxygen uptake; Ẇ: Steady power production during walking in kilocalories per minute; *ϵ*_*net*_: Net mechanical efficiency of walking; 1RM: One repetition maximum.

## Author’s contributions

GM, JH, JH and JH designed the study. JH and GEN recruited patients, performed testing and other data acquisition. GM and JH undertook the statistical analysis and JH wrote the first draft of the paper. All authors have contributed to and have approved the final manuscript.

## Competing interests

The authors declare that they have no competing interests.
